# Lopinavir inhibits insulin signaling by promoting protein tyrosine phosphatase 1B expression

**DOI:** 10.3892/etm.2014.1826

**Published:** 2014-07-04

**Authors:** TAKATOSHI KITAZAWA, YUSUKE YOSHINO, SATOSHI SUZUKI, ICHIRO KOGA, YASUO OTA

**Affiliations:** 1 Department of Medicine, Teikyo University School of Medicine, Tokyo 173-8605, Japan; 2 Department of Pulmonary Medicine, Graduate School of Medicine, The University of Tokyo, Tokyo 113-8655, Japan

**Keywords:** lopinavir, darunavir, insulin, protein tyrosine phosphatase 1B

## Abstract

Treatment with antiretroviral therapy, including protease inhibitors (PIs), may result in metabolic side-effects, for example insulin resistance. The aim of the present study was to investigate the mechanism of the dysregulation of insulin signaling by two PIs, lopinavir and darunavir, by analyzing changes in the expression or activity of proteins associated with insulin signaling. 3T3-L1 preadipocytes were pretreated with lopinavir or darunavir for 48 h and then stimulated with insulin for 30 min. The cell lysates were subjected to western blotting with anti-phospho-insulin receptor substrate (IRS) 1, anti-IRS1, anti-suppressor of cytokine signaling (SOCS) 1, anti-SOCS3 and anti-protein tyrosine phosphatase (PTP) 1B antibodies and to immunoprecipitation with anti-IRS1 antibody. Translocation of glucose transporter 4 (GLUT4) following treatment with lopinavir or darunavir was observed using immunofluorescence. While GLUT4 was recruited to the cellular membrane in control adipocytes following insulin stimulation, it was diffusely distributed in the cytosol in lopinavir-treated adipocytes. In darunavir-treated adipocytes, GLUT4 was mainly recruited to the cellular membrane, but some GLUT4 remained in the cytosol. After insulin stimulation, IRS1 was tyrosine-phosphorylated to a greater extent in control adipocytes compared with darunavir-treated adipocytes. Tyrosine phosphorylation of IRS1 was inhibited in lopinavir-treated adipocytes. The expression of PTP1B was upregulated in adipocytes pretreated with the PIs, particularly lopinavir, compared with those pretreated with a vehicle control. The degree of regulation in insulin signaling differs between lopinavir and darunavir. One mechanism by which lopinavir regulates insulin signaling is by the promotion of PTP1B expression.

## Introduction

In recent years, significant advances in HIV treatment have been made towards reducing mortality in HIV-infected patients (1). However, patients treated with antiretroviral therapy, including protease inhibitors (PIs), develop metabolic side-effects, including hyperlipidemia, insulin resistance, lipoatrophy and lactic acidosis (2).

The molecular mechanism of PI-induced insulin resistance has not yet been elucidated. Previous studies have suggested that PI-induced insulin resistance and diabetes are associated with the inhibition of glucose transporter 4 (GLUT4) translocation (3,4) and that lopinavir inhibits the phosphorylation of insulin receptor substrate (IRS) (5).

A previous study demonstrated that increased inflammation in adipose tissue is a prominent mechanism of insulin resistance (6). In addition, increased levels of pro-inflammatory cytokines secreted from adipose tissue activate a variety of cellular events that impede insulin action in adipose tissue (7). Suppressor of cytokine signaling (SOCS) 1 is one of the main molecules involved in inflammatory signaling; it has Src homology 2 domains, interacts with Janus kinase and inhibits the kinase activity of inflammatory cytokines (8). Among the SOCS family members, SOCS1 and SOCS3 induce insulin resistance by inhibiting the phosphorylation of IRS (9).

The insulin signal transduction system also includes protein tyrosine phosphatases (PTPs), enzymes that dephosphorylate tyrosine kinases. PTP1B is a negative regulator that has an important role in the metabolic system, immune system and oncogenesis (10).

In the present study, it was hypothesized that PIs affect insulin signaling by regulating the expression of SOCS or PTPs. Therefore, the aim of the study was to investigate the mechanism of the dysregulation of insulin signaling induced by lopinavir and darunavir, which are widely used protease inhibitors. In particular, changes in the activities of SOCS and PTP1B caused by PI treatment were analyzed.

## Materials and methods

### Materials

Lopinavir and darunavir were purchased from Toronto Research Chemicals Inc. (Toronto, Ontario, Canada) and dissolved in ethyl acetate and methanol, respectively. Since the levels of IRS1 expression and IRS1 phosphorylation by insulin were comparable in preliminary experiments, methanol was used as a vehicle control in the following experiments. Insulin from bovine pancreas was obtained from Sigma-Aldrich (St. Louis, MO, USA). The primary antibodies used were anti-phospho (Ser307)-IRS1 and anti-IRS1 antibodies (Upstate Biotechnology Inc., Lake Placid, NY, USA), and anti-SOCS1, anti-SOCS3 and anti-PTP1B antibodies (Santa Cruz Biotechnology, Santa Cruz, CA, USA). The phospho-tyrosine-specific monoclonal antibody 4G10 (Upstate Biotechnology, Darmstadt, Germany) was used in the immunoprecipitation assay.

### Cell culture, pretreatment with PIs and insulin stimulation

3T3-L1 preadipocytes were obtained from the American Type Culture Collection (Manassas, VA, USA) and cultured and maintained as previously described (11). Differentiated adipocytes were obtained by plating preadipocytes in differentiation medium containing insulin, dexamethasone, isobutyl methyl xanthine and a thiazolidinedione (AM-1; DS Pharma Biomedical Co. Ltd., Osaka, Japan) for an additional 7 days.

Adipocytes were pretreated with PIs by adding 30 μM lopinavir, 30 μM darunavir or a vehicle control (0.1% ethyl acetate or 0.1% methanol, respectively) for 48 h. Following PI pretreatment, adipocytes were stimulated with 100 nM of insulin for 30 min.

### Western blotting and immunoprecipitation

Following insulin stimulation, ice-cold phosphate-buffered saline (PBS) was added, and cells were lysed with NP-40 lysis buffer containing 1% Nonidet P-40, 25 mM Tris-HCl (pH 7.5), 150 mM sodium chloride, 1 mM EDTA, 5 mM sodium fluoride, 1 mM sodium orthovanadate, 1 mM leupeptin and 1 mM phenylmethylsulfonyl fluoride. The lysates were resuspended in loading buffer as described by Laemmli (12). Sodium dodecyl sulfate-polyacrylamide gel electrophoresis (SDS-PAGE) was performed with 10–12% (w/v) acrylamide gels (12). The separated proteins were transferred onto a nitrocellulose membrane for immunoblotting. The membrane was blocked in blocking buffer for 1 h, then incubated with a primary antibody, followed by a horseradish peroxidase (HRP)-labeled secondary antibody (Sigma-Aldrich). The protein bands were then visualized using a chemiluminescence reagent (Immobilon Western chemiluminescent HRP Substrate; Millipore, Billerica, MA, USA).

For the immunoprecipitation studies, cell lysates were mixed with 4 μg anti-IRS1 antibody for 1 h. Cell lysates were then mixed with protein G-coupled Sepharose beads (GE Healthcare UK Ltd., Little Chalfont, UK) and rotated for 1 h at 4°C. The beads were washed 3 times with ice-cold NP-40 lysis buffer and the precipitated proteins were boiled for 5 min and eluted with loading buffer. SDS-PAGE and western blot analysis were performed with 4G10 antibody as described above.

### Immunodetection of GLUT4

The 3T3-L1 adipocytes grown on coverslips were pretreated with protease inhibitors and stimulated with insulin as described above. Following insulin stimulation, cells were placed on ice, washed twice in ice-cold PBS and fixed with 4% (w/v) paraformaldehyde in PBS for 15 min. The reaction was quenched with 0.1 M glycine in PBS for 10 min. Samples were then blocked with PBS containing 5% bovine serum albumin for 10 min and incubated with 5 μg/ml anti-GLUT4 antibody (LifeSpan Biosciences, Inc, Seattle, WA, USA) for 16 h at 4°C and for 45 min with secondary Alexa 594-conjugated anti-mouse immunoglobulin antibodies (Molecular Probes, Inc, Eugene, OR, USA) at room temperature. Coverslips were washed twice with PBS and mounted with Dako mounting solution (Dako Japan, Tokyo, Japan). Images were captured using a fluorescence microscope (ECLIPSE TE2000-U; Nikon, Kanagawa, Japan) by argon laser (excitation, 594 nm) at room temperature with a ×40 objective lens at the same setting.

## Results

### GLUT4 recruitment to the plasma membrane is inhibited by lopinavir and darunavir

**Figure 1:**
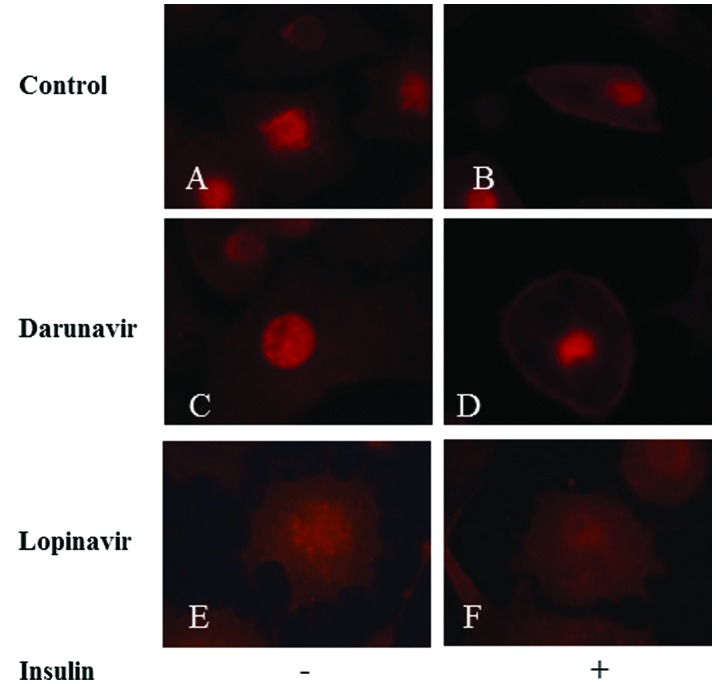
Insulin-induced recruitment of GLUT4 to the plasma membrane was inhibited in adipocytes pretreated with lopinavir or darunavir. Mature 3T3-L1 adipocytes were pretreated with darunavir (30 μM), lopinavir (30 μM) or a control vehicle, and were then stimulated with or without 100 nM of insulin for 30 min. The localization of GLUT4 was determined by immunofluorescence. Control adipocytes in the (A) absence and (B) presence of insulin stimulation; darunavir-pretreated adipocytes in the (C) absence and (D) presence of insulin stimulation; lopinavir-pretreated adipocytes in the (E) absence and (F) presence of insulin stimulation. GLU4, glucose transporter 4.

3T3-L1 adipocytes were pretreated with lopinavir, darunavir or a vehicle control and were stimulated with insulin for 30 min. GLUT4 localization was then observed using immunofluorescence. In the control adipocytes, GLUT4 was localized diffusely in the cytosol without insulin stimulation (Fig. 1A) and then translocated to the plasma membrane following insulin treatment (Fig. 1B). In adipocytes treated with darunavir, the distribution of GLUT4 was similar to that in the control cells in the absence of insulin (Fig. 1C). However, following insulin stimulation, GLUT4 was recruited to the cellular membrane, but some GLUT4 was observed to remain in the cytosol (Fig. 1D). However, in lopinavir-treated adipocytes, only a small quantity of GLUT4 was recruited to the plasma membrane following insulin treatment (Fig. 1E and F). These results indicate that PIs, in particular lopinavir, inhibit insulin-induced GLUT4 recruitment to the plasma membrane.

### Lopinavir inhibits IRS1 phosphorylation

**Figure 2:**
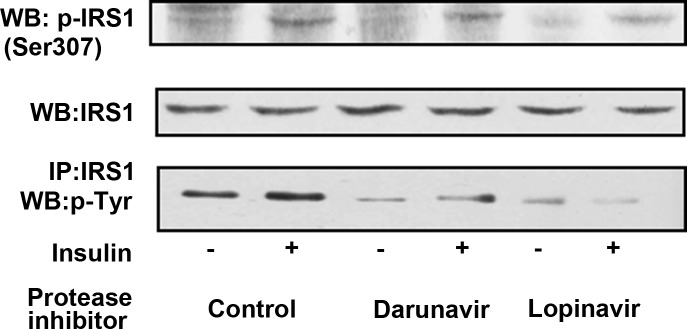
Insulin-induced IRS1 phosphorylation was inhibited in adipocytes pretreated with lopinavir. Mature 3T3-L1 adipocytes were pretreated with darunavir (30 μM), lopinavir (30 μM) or a control vehicle, and then were stimulated with or without 100 nM of insulin for 30 min. The cell lysates were resolved using SDS-PAGE and visualized by immunoblotting with a 1:2,000 dilution of anti-phospho (Ser307)-IRS1 antibody (top panel) or with a 1:2,000 dilution of anti- IRS1 antibody (middle panel). In addition, the cell lysates were immunoprecipitated with IRS1 antibody, and the immunoprecipitated proteins were resolved using SDS-PAGE and visualized by immunoblotting with a 1:2,000 dilution of anti-phospho-tyrosine (4G10) antibody (bottom panel). IRS1, insulin receptor substrate 1; SDS-PAGE, sodium dodecyl sulfate-polyacrylamide gel electrophoresis; WB, western blot; IP, immunoprecipitation.

3T3-L1 adipocytes were pretreated with a control vehicle, lopinavir or darunavir, and then stimulated with insulin for 30 min. The levels of IRS1 expression did not differ in control adipocytes and adipocytes treated with lopinavir or darunavir prior to and following insulin stimulation (Fig. 2). In the absence of insulin stimulation, IRS1 at Ser307 was not phosphorylated in the control adipocytes or the lopinavir- or darunavir-pretreated adipocytes. However, following insulin stimulation, IRS1 at Ser307 was phosphorylated in control adipocytes, and it was phosphorylated in a similar manner in darunavir- and lopinavir-pretreated adipocytes. In addition, IRS1 was also tyrosine-phosphorylated following insulin stimulation in control adipocytes. However, in darunavir-treated adipocytes, tyrosine phosphorylation of IRS1 was reduced. Furthermore, it was almost completely inhibited in lopinavir-pretreated adipocytes.

### Lopinavir and darunavir do not affect SOCS expression

**Figure 3:**
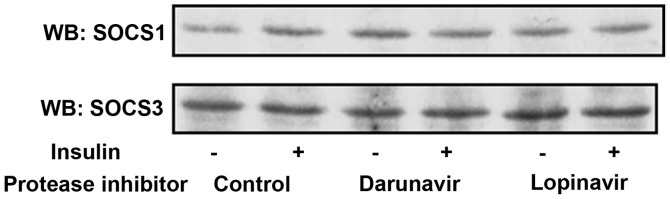
SOCS were not upregulated following stimulation of insulin in adipocytes pretreated with lopinavir or darunavir. Mature 3T3-L1 adipocytes were pretreated with darunavir (30 μM), lopinavir (30 μM) or a control vehicle, and were then stimulated with or without 100 nM of insulin for 30 min. The cell lysates were resolved using SDS-PAGE and were visualized by immunoblotting with an anti-SOCS1 antibody (upper panel) or with an anti-SOCS3 antibody (lower panel). SOCS, Suppressor of cytokine signaling; SDS-PAGE, sodium dodecyl sulfate-polyacrylamide gel electrophoresis; WB, western blot.

SOCS family members are negative regulators of insulin signaling. The expression of SOCS1 and SOCS3 was compared between control adipocytes and PI-pretreated adipocytes. In the absence and presence of insulin stimulation, the expression levels of SOCS1 or SOCS3 did not change among the cells (Fig. 3). Analysis of the results from the immunoprecipitation assay demonstrated that neither SOCS1 nor SOCS3 was associated with IRS1 in the control adipocytes and PI-treated adipocytes prior to and following insulin stimulation (data not shown). These results indicate that PIs did not influence the expression of SOCS1 and SOCS3 and were not associated with them.

### Lopinavir promotes PTP1B expression

**Figure 4:**
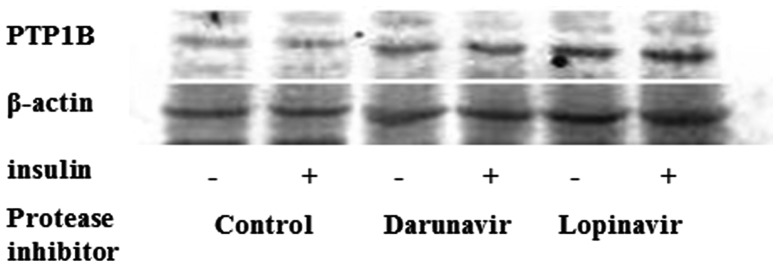
Insulin stimulation leads to up-regulation of PTP1B in adipocytes pretreated with lopinavir. Mature 3T3-L1 adipocytes were pretreated with darunavir (30 μM), lopinavir (30 μM) or a control vehicle, and then were stimulated with or without 100 nM of insulin for 30 min. The cell lysates were resolved using SDS-PAGE and visualized by immunoblotting with anti-PTP1B antibody (upper panel) or anti-β-actin antibody as an internal control (lower panel). PTP, protein tyrosine phosphatase; SDS-PAGE, sodium dodecyl sulfate-polyacrylamide gel electrophoresis.

The levels of PTP1B expression were compared among control adipocytes and PI-pretreated adipocytes. The expression levels of PTP1B were enhanced in adipocytes pretreated with protease inhibitors, in particular lopinavir (Fig. 4). However, no significant differences were identified in the levels of PTP1B expression among the adipocytes prior to and following insulin treatment.

## Discussion

To investigate the regulation of insulin signaling, 3T3-L1 adipocytes and 30 μM lopinavir and darunavir were used. The mean C_min_ values of lopinavir and darunavir are 4.6 μg/ml (7.3 μM) and 1.8 μg/ml (3.1 μM), respectively, and the C_max_ values of lopinavir and darunavir are 10.0 μg/ml (15.9 μM) and 8.2 μg/ml (13.8 μM) (13,14). Glucose uptake is inhibited with 10–100 μM PIs (3). The concentration of lopinavir and darunavir that was used in the present study is consistent with the dosage used in clinical settings.

PIs mediate their antiviral effect by cleaving HIV protease, the pol gene product (15). Protease inhibitors have several targets in insulin signaling (16). In the present study, it was found that PIs upregulate PTP1B expression. This target is considered to be critical since the levels of PTP1 expression are consistent with the degree of inhibition of IRS1 tyrosine phosphorylation and GLUT4 translocation that are associated with insulin resistance in the clinical setting.

Indinavir (100 μM) has been shown to significantly inhibit GLUT4 activity in *Xenopus* oocytes (3). In the present study, the effects of lopinavir and darunavir on insulin resistance were investigated by analyzing the changes of GLUT4 recruitment to the plasma membrane using immunofluorescence. However, translocation of GLUT4 was not investigated for other PIs, including lopinavir and darunavir, by immunofluorescence in previous studies. The immunofluorescence results in the present study following treatment with lopinavir or darunavir appear to be consistent with previous results.

IRS1 phosphorylation, which is activated by insulin signaling, was also investigated in this study. Increased IRS-1 phosphorylation of serine and threonine residues, in particular Ser307, contributes to the defective IRS-1 tyrosine phosphorylation in insulin-resistance (17). Ser307 phosphorylation was not observed to be significantly enhanced in the PI-treated adipocytes. However, tyrosine phosphorylation of IRS-1 was inhibited in adipocytes treated with PIs, in particular with lopinavir. Ismail *et al* (18) demonstrated that pretreatment with indinavir induced a significant reduction in insulin-induced tyrosine phosphorylation of IRS-1, and these results were consistent with the results from the present study. This study focused on PTP1B, which inhibits IRS1 tyrosine phosphorylation, and it was found that PTP1B expression was enhanced in the presence of PIs. Following insulin binding, the insulin receptor tyrosine kinase becomes activated and phosphorylates IRS1 protein on tyrosine residues, which serve as binding sites for phosphatidylinositol 3-kinase (PI3K). PI3K catalyzes the phosphorylation of phosphatidylinositol at the 3′-position and generates 3′-phophatidylinositol products. Subsequent signaling pathways induce the translocation of the glucose transporter GLUT4. Enhancement of PTP1B expression may lead to the dephosphorylation of tyrosine residues on several substrates, including IRS-1, resulting in the downregulation of insulin signaling (19). Ben-Romano *et al* (20) demonstrated that a direct inhibitory effect on insulin-induced glucose uptake occurs following a specific interaction of protease inhibitors with GLUT4, whereas prolonged exposure to nelfinavir interferes with PKB phosphorylation. In a study by Schütt *et al* (21), impaired insulin secretion by nelfinavir or saquinavir was found to be associated with decreased insulin-induced IRS-1 phosphorylation, although amprenavir and indinavir had no effect on insulin secretion. Ismail *et al* (18) reported that the levels of PTP1B were not altered in adipocytes treated with indinavir, which is not in accordance with the results from the present study and the reason for this has yet to be elucidated. However, it may be hypothesized that the PIs may affect multiple sites in insulin signaling and that, therefore, the regulatory effects may differ among PIs.

In the present study, lopinavir had a stronger inhibitory effect on insulin signaling compared with darunavir. This is the first study, to the best of out knowledge, to compare insulin sensitivity between darunavir and lopinavir. In a previous study comparing insulin sensitivity between atazanavir and lopinavir *in vitro* and clinically, the area under the curve of glucose increased significantly with lopinavir/ritonavir, but not with atazanavir/ritonavir during oral glucose tolerance tests (22). In another study investigating HIV-negative healthy volunteers receiving darunavir/ritonavir or atazanavir/ritonavir it was found that the glucose parameters did not differ between the two groups (23). Björnholm *et al* (24) reported that reduced insulin-stimulated IRS-1 tyrosine phosphorylation led to impaired insulin-induced glucose transport in the skeletal muscle of obese diabetic patients. Assuming that there was no difference in the impact of boosted ritonavir in insulin signaling among lopinavir, atazanavir, and darunavir, this suggests that the results from the present study are consistent with these clinical results.

Although lopinavir and darunavir inhibited insulin signaling in adipocytes, lopinavir had a stronger inhibitory effect on the recruitment of GLUT4 to the cellular membrane and the tyrosine phosphorylation of IRS-1 compared with darunavir.
